# A Comparison of the Vertical Transmission of High- and Low-Virulence Nucleopolyhedrovirus Strains in *Lymantria Dispar* L.

**DOI:** 10.3390/insects11070455

**Published:** 2020-07-20

**Authors:** Yuriy B. Akhanaev, Irina A. Belousova, Darya A. Lebedeva, Sergey V. Pavlushin, Vyacheslav V. Martemyanov

**Affiliations:** 1 Laboratory of Ecological Physiology, Institute of Systematics and Ecology of Animals SB RAS, Frunze str. 11, Novosibirsk 630091, Russia; belousova_i@yahoo.com (I.A.B.); lebedeva_dasha2011@mail.ru (D.A.L.); sergey-pavlushin@mail.ru (S.V.P.); 2 Reshetnev Siberian State University of Science and Technology, Krasnoiarskii rabochii av. 31, Krasnoyarsk 630091, Russia

**Keywords:** Lymantria dispar, baculovirus, vertical transmission, covert infection, reactivation

## Abstract

Baculoviruses can persist in insect host organisms after infection and may be vertically transmitted to the next generation, in which they may be reactivated. The goal of the present study was to compare the efficiency of the vertical transmission of high- and low-virulence strains and the subsequent reactivation of Lymantria dispar multiple nucleopolyhedrovirus (LdMNPV) in the offspring of *Lymantria dispar* L. adults who survived after viral infection. As a result of parental infection, the fecundity of survived females, pupae weight, and fertility were significantly different compared to the untreated insects. However, differences in these parameters between high- and low-virulence strains were not observed. The prevalence of virus strains in the offspring measured by quantitative polymerase chain reaction also did not differ. When the larvae reached the fourth instar, they were starved to activate the vertically transmitted virus. The frequency of virus activation in the experiment was not dependent on the virulence of the virus strains. These results are helpful for understanding the strategy of virus survival in nature and for the selection of the most effective strains with transgenerational effects in the years following pest treatment.

## 1. Introduction

The causes of cycles in insect pest populations have long been a major focus in ecological research [1,2]. Among the many factors affecting the cycles of insect pest populations, baculoviruses occupy an important role [3,4]. Specifically, baculoviruses are important natural enemies of many lepidopteran species and have been developed as biological control agents for a range of pests in agriculture and forestry [5,6,7]. They are generally highly pathogenic and infect host larvae during feeding [8]. Nucleopolyhedrovirus (NPV) diseases involve the production of millions of occlusion bodies (OBs) containing occlusion-derived virions [9] that are visible under a light microscope. Upon host death, liquefaction occurs and the external cuticle of the cadaver ruptures, resulting in the liberation of OBs. This leads to the contamination of plant substrates (including leaves), where OBs may be horizontally transmitted to healthy larvae [10]. Virus-induced mortality is observed at the larval and pupal stages and is never observed at the adult stage.

It is known that baculoviruses can be transmitted not only horizontally but also vertically. Horizontal transmission is thought to be a major route of baculovirus transmission [11]. Vertical transmission of baculovirus occurs through the transovarial and transovum routes [10,12]. Transovarial transmission involves virus passage to offspring embryos within the eggs, whereas the transovum route involves contamination of the egg surface with viral particles during the oviposition process that infect the neonate larvae when they ingest the chorion during hatching. In order to methodically divide means of vertical transmission, the surfaces of eggs are usually decontaminated using disinfectants [13,14]. It was found that the external disinfection of eggs reduces the virus prevalence of offspring of many insects [12] and is regularly used during insect rearing to reduce the risk of epizootics of baculovirus in laboratory colonies. The development of technologies such as standard polymerase chain reaction (PCR) and quantitative PCR (qPCR) has allowed the role of transovarial transmission of baculovirus and its influence on the dynamics of the host population to be evaluated [15,16,17,18].

The horizontal and vertical types of transmission strategies generally exclude one another because a virus with high virulence kills the host prior to the adult stage. This mean that the ability for vertical transmission will be selected during natural selection in high virulent strains [4]. For this, vertically transmitted strains should possess low virulence to be effectively transmitted to the next host generation by way of surviving adults. Low virulence of the virus is associated with a covert infection that does not have any signs of lethal NPV disease [12,17]. There are two types of covert infection: (i) persistent infection that involves low levels of virus replication in the host cell and (ii) latent infection wherein the virus is essentially dormant. The first type of covert infection has been proven for baculoviruses [15,19]. The second type has not been proven for baculoviruses, although it has been demonstrated for other viruses [20]. These types of covert infections can be activated in virus-harboring hosts and pass to overt infections by exposure to physiological stresses such as high host densities, high relative humidity, starvation, food quality, the presence of other pathogens leading to death, and horizontal transmission [21,22,23,24,25,26]. The presence of a covert infection was proposed to explain the spontaneous outbreaks of baculovirus epizootics in apparently healthy insects [3,27]. Nevertheless, how the formation of covert infection occurs in insects and the duration in which a covert infection can be activated by stress factors remain poorly understood. 

Lymantria dispar multiple nucleopolyhedrovirus (LdMNPV) is a highly pathogenic, host-specific *Alphabaculovirus* that has been used as the basis of biological insecticides for the control of the gypsy moth, *Lymantria dispar* L. (Lepidoptera: Erebidae) [28,29]. To date, many different geographic strains of LdMNPV have been genetically characterized [30,31,32] and biological activity against gypsy moth larvae has been quantified [10,33,34,35,36]. It has been found that strains may differ significantly in terms of virulence. For example, the strains of LdMNPV (LdMNPV-45/0) isolated from Northern American populations of *L. dispar* possess a more than 10-fold higher potency over three LdMNPV (LdMNPV-27/0) strains isolated from a continental Asian (Western Siberia) host population [36]. In addition, a previous study demonstrated that surviving *L. dispar* individuals that consumed OB could vertically transmit the virus to offspring and were detected in the following two generations by PCR [37]. We hypothesized that a low-virulence strain has an advantage in its capability of being transmitted vertically to the next generation when compared to a highly virulent strain. The primary goal of the present study is to estimate the prevalence of the vertical transmission of strains that significantly differed in their virulence after virus inoculation of parents. We also considered the capability of these strains to be activated as an overt infection in the next generation using starvation as the triggering factor for virus activation [23,24]. Additionally, we compared the debilitating effects of virus strains on the performance of parents that survived after treatment.

## 2. Materials and Methods

### 2.1. Insects and Virus

The egg masses of diapausing insects were collected in the Novosibirsk region (Western Siberia; 54.33° N, 81.13° E) in autumn 2016 and stored in a refrigerator at 2 °C. The population of *L. dispar* was at the rising phase of population dynamics. The egg masses were mixed, and the result used as a single stock for treatment (more details in Section 2.2). The stock was estimated on the prevalence of covert LdMNPV infection in the egg masses of host populations by qPCR. Ten pooled samples of *L. dispar* eggs containing 10 eggs per pool were randomly chosen from the mixed egg masses for the analysis. In total, 100 eggs were analyzed. Total DNA was extracted from each egg using the phenol–chloroform method [38] with some modification. To eliminate possible contamination of the egg surface of virus by transovum route we placed each egg in a microcentrifuge tube (1.5 mL) with 1 mL of 1% sodium hypochlorite for 10 minutes followed by washing with sterile water [13]. In addition, the eggs were kept under ultraviolet light for the same time with shaking [14] for the degradation of viral DNA on the eggs’ surface. Next, each egg was mechanically homogenized with a pestle in a lysis solution containing guanidine isothiocyanate for DNA extraction (kit C-8879; VECTOR-BEST, Russia). A fragment of the polyhedrin gene was used as the target sequence for detecting the presence of LdMNPV DNA in the samples. The amplification reactions for the analysis of the gypsy moth NPV polyhedrin gene contained 50 nM of primer 1, 50 nM of primer 2, DNA at 500 ng/25 μL at the final volume, and the reaction mixture HS-qPCR Mix SYBR (Biolabmix, Novosibirsk, Russia). The reaction conditions were five minutes at 95 °C, 40 cycles of 30 s at 95 °C, and one minute at 60 °C, with a melting curve of 60 °C to 95 °C. The primers for the gypsy moth NPV polyhedrin gene were LdPH1268-F 5’-GCACTTCCTCAACTCGGTCA-3’ and LdPH1394-R 5’-CGTTTAGTACGCCGGTCCTT-3’ (Primer-BLAST; National Institutes of Health, Bethesda, MD, USA). Viral DNA detection was conducted using SYBR Green on a CFX96 (Bio-Rad Laboratories, Hercules, CA, USA). The sensitivity of the reaction was assessed using 2-fold serial dilutions of pure virus DNA with a known concentration. The most reliable and repeatable limit of detection was equivalent to 3 viral genome copies of LdMNPV. qPCR detection did not reveal the amplification of LdMNPV-specific DNA fragments in any tested samples of stock *L. dispar* eggs. Thus, the mixed stock of *L. dispar* was considered to be free from endogenous virus or to have an extremely low virus prevalence.

In spring 2017, the eggs of the stock were sterilized using 1% sodium hypochlorite. Then, the eggs were placed in a Petri dish with 100 eggs each. The Petri dishes were kept under thermostat at a constant temperature (28 °C) for 48 hours for egg hatching. Next, neonate *L. dispar* were placed in plastic containers (100 individuals/20 L container) and fed with the leaves of cut branches of silver birch *Betula pendula* Roth. The insects were reared under laboratory conditions at a constant temperature (23 °C) and in a natural daylight regime. Then, insects were used for the treatments.

### 2.2. Virus Treatments and the Life-History Traits of Surviving Parents

Larvae (1 day after fourth instar molting) were infected with two strains of LdMNPV from the collection of the Laboratory of Ecological Physiology of the Institute of Systematics and Ecology of Animals (Novosibirsk, Russia): strain 45/0 (Springfield, MA, USA; 42.15° N, 72.49° W), kindly given by Dr John Podgwaite (USDA Forest Service) and strain 27/0 (isolated in the Novosibirsk region, Russia; 55.14° N, 75.52° E). 

To initiate infection, groups of 250 fourth instar larvae were allowed to eat leaf discs (dia = 8 mm) containing a dried drop of suspension of 4 × 10^4^ OBs/disc for LdMNPV-45/0 and 2 × 10^5^ OBs/disc for LdMNPV-27/0 as described in detail by Akhanaev et al., 2017 [39]. The doses used significantly differed for the low- and high-virulence strains. Based on previous studies, these doses of OBs were designed to kill approximately 50% to 60% of the insects. One additional group of larvae consumed leaf disc with water as untreated insects. Only larvae that entirely consumed their leaf discs within 12 hours were included in the subsequent bioassay. To exclude horizontal transmission, the infected larvae were reared individually in plastic containers (125 mL) until death or pupation.

Once the adults emerged from surviving pupae, they were moved into plastic containers (1000 mL) (1♀:1♂) with filter paper for oviposition. Males and females from the same viral strain treatment were used. After mating and oviposition, egg masses were collected and marked to indicate them as belonging to parental pairs of adults. The following life-history traits were compared for those adults that survived OB treatment between treated and untreated insects: larval development time (from egg hatching to pupation), pupal weight, duration of the pupal stage, sex ratio, fecundity, and fertility. Since females of *L. dispar* have clear sexual dimorphism (e.g., females have one additional larval stage, different weight), the analysis was performed separately between males and females. The collected egg mass was kept at room temperature for two months for postembryonic development. Then, the eggs were moved to a refrigerator at 2 °C for winter diapause. In the following year, the offspring were used for estimating success transmission of the virus. 

### 2.3. Estimation of the Transmitted Virus in Offspring

To estimate the prevalence of transmitted LdMNPV infection in the offspring, we used the qPCR technique. For this, we randomly selected 5 eggs from each egg mass laid by females that survived after infection. Total DNA was extracted from each egg individually (*n* = 320) and analyzed as described in Section 2.1. 

Separately, another pool of eggs from the same egg masses described in the previous paragraph was used to examine the capability of vertically transmitted LdMNPV infection to its activation. After overwintering, each egg mass from surviving parents was sterilized for the elimination of LdMNPV from egg surfaces using disinfectant (see above). The offspring were reared under laboratory conditions. When larvae reached the fourth instar, they were exposed to starvation to test for possible activation of the transmitted virus. Previously we showed that starvation is an effective stress-factor for the activation of covert to overt infection [23,24]. To do this, the larvae of second generation (i.e., the next generation after virus infection) were placed in individual plastic containers (125 mL) and the offspring of each family were divided into two lines. The first line continued to feed on leaves of the host plant until pupation (without starvation), while the second line of larvae was starved for three days to activate the vertically transmitted virus. In summary, 375 larvae of LdMNPV-45/0 and 200 larvae of LdMNPV-27/0 were starved and the same amount were used as untreated insects. After three days of starving, feeding was resumed. Mortality was registered daily until all insects either died or pupated. All dead larvae were checked for viral disease using light microscopy (Axioscope 40 Carl Zeiss, Germany) to determine the presence of OBs. 

### 2.4. Statistical Analysis

Mortality of host treatments was analyzed using the generalized linear model with a binomial distribution and a logit link function (GLM). As no untreated insects died of virus infection, no correction factors needed to be applied. Non-viral deaths, predominantly fungal, did occur at a very low level (<1%) late in the assay. Data on the influence of the virus on the life history of surviving parents and reproduction were evaluated with the Shapiro–Wilk W test. Non-normally distributed data were analyzed with the Kruskal–Wallis test, followed by Dunn’s post hoc test. Normally distributed data were analyzed by one-way ANOVA, followed by Tukey’s post hoc test (only for data of fertility). Data of median development time of pupae were analyzed using a nonparametric Mann–Whitney U test between virus strains.

A comparison of effects test of the vertically transmitted virus in the next generation between low- and high virulence strain treatments was conducted by GLM. Data were analyzed using the program PAST 3.0 [40] and statistical programming language R [41].

## 3. Results

### 3.1. Virus Treatment of the Stock Lymantria Dispar Population and the Life-History Traits of Surviving Parents

Host survival was severely reduced by baculovirus infection (χ^2^ *=* 140.1, df = 1, *p* < 0.0001). The percentages of NPV mortality were 49.2% for LdMNPV-45/0 and 63.5% for LdMNPV-27/0. We observed that the mortality caused by LdMNPV-45/0 was lower and the difference was statistically significant as compared with LdMNPV-27/0 (χ^2^ = 146.8, df = 1, *p* < 0.0001). This difference was the result of different viral loads for different strains in which we tried to equalize the induced mortality of the hosts. All dead larvae were characterized by typical signs of polyhedrosis following infection by both viral strains.

The larva development time was longer for larvae of both sexes that survived the OB-treated when compared to untreated insects (males: H_2, 271_ = 74; *p* < 0.001, females: H_2, 299_ = 71.2, *p* < 0.001, Table 1). Moreover, a significant difference was found in the duration of larval development between strains (Dunn’s test, *p* < 0.05), although differences in absolute values were as low as 1 day. The duration of the pupal stage was not significantly affected for both sexes by OB treatment (male: Mann–Whitney U = 2692.5, *n* = 164, *p* = 0.923; female: Mann–Whitney U = 2624, *n* = 165, *p* = 0.065). Pupal weight was significantly higher among the survivors of OB treatment when compared to in the untreated insects (H_2, 557_ = 61.1, *p* < 0.001). The surviving pupae after LdMNPV-27/0 were heavier than those surviving after LdMNPV-45/0 infection (Dunn’s test, *p* < 0.05). The sex ratio of survived adults did not differ significantly between strains (*p* > 0.05), although the proportion of males was lower in the untreated insects when compared with the OB-treated insects (*p* < 0.05). The fecundity of parental females was reduced by up to 30% in OB-treated insects when compared to the untreated insects (H_2, 54_ = 22.1, *p* < 0.001), independently of virus strains (Dunn’s test, *p* > 0.05). Finally, fertility as measured by the proportion of eggs that successfully hatched was significantly lower in OB treatments than in the untreated insects (F_2, 44_ = 11.2, *p* < 0.05) and did not differ between virus strains (Tukey’s test, *p* > 0.05, Table 1).

### 3.2. Estimation of the Transmitted Virus in Offspring

**Figure 1:**
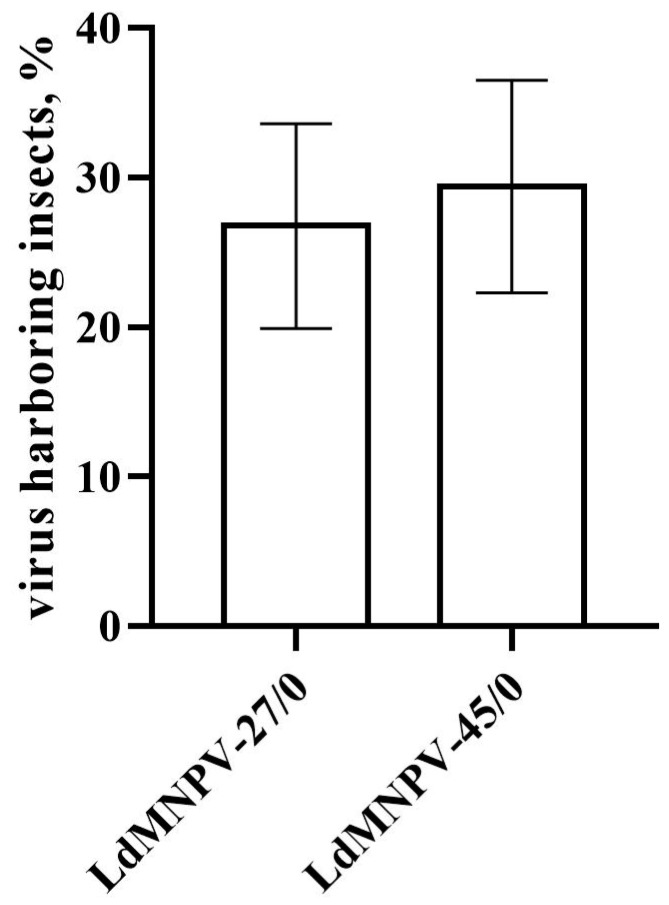
Prevalence of covert viral infection in *Lymantria dispar* eggs after vertical transmission of low (LdMNPV-27/0) and high (LdMNPV-45/0) virulent strains via one host generations.

The prevalence of the vertically transmitted virus in eggs varied depending on the family of survivors (χ^2^ = 69.8, df = 26, *p* < 0.001). The mean percentage of offspring positive for viral DNA did not exceed 30% in the OB-treated insects and did not differ significantly between virus strains (χ^2^ = 0.35, df = 1, *p* = 0.552, Figure 1).

**Figure 2:**
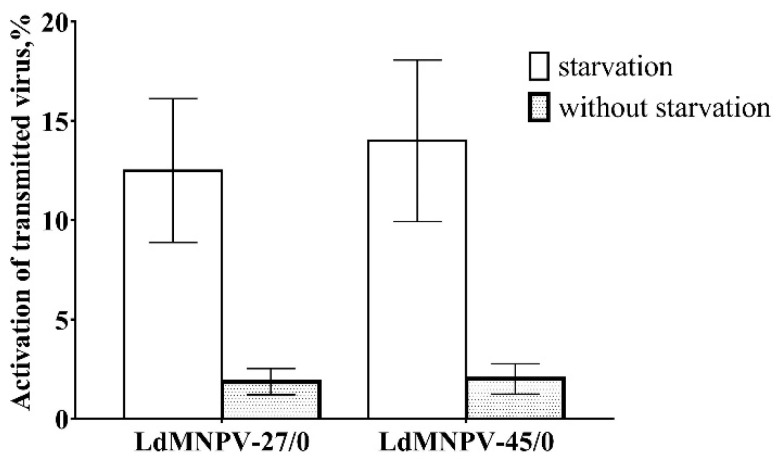
The frequency of activation of vertically transmitted virus in offspring of *Lymantria dispar* parents infected by low (LdMNPV-27/0) and high (LdMNPV-45/0) virulent strains.

According to our model, the starvation activated vertically transmitted virus from OB-treated parents in the next generation (χ^2^ = 30.6, df = 1, *p* < 0.001, Figure 2) while starvation did not cause LdMNPV-induced mortality in the untreated insects. However, frequency activation of the virus was not different between virus strains (χ^2^ = 0.00, df = 1, *p* = 0.987). The interaction between virus strain and starvation was non-significant (χ^2^ = 0.06, df = 1, *p* = 0.802). In parallel, we observed a low frequency of activation of the virus in the OB treatment insects reared without starvation, which was not significantly different among virus strains (χ^2^ = 0.01, df = 1, *p* = 0.911, Figure 2). Importantly, there was a low number of cadavers seen during starving, although virus activation mainly occurred after feeding was resumed.

When we calculated the frequency of vertically transmitted virus activation in relation to virus-harboring larvae (not the total number of larvae), we observed more marked differences in activation rates of compared strains: 81.8% in the LdMNPV-27/0-treated insects and 55.7% in the LdMNPV-45/0-treated insects. 

## 4. Discussion

Studies of vertical baculovirus transmission in *L. dispar* have previously been conducted with conflicting results. For example, Murray et al. (1991) [42] reported that they did not record the transmission of the virus from parents to offspring. Elsewhere, Shapiro et al. (1987) [43] observed the death of insects from spontaneous polyhedrosis in the first generation at prevalence ranging from 4.7% to 11.5%. The authors suggested the insect mortality from polyhedrosis among the offspring of infected parents was the result of the vertical transmission of the virus. Later, other authors and the results of the current study also noted the transmission of LdMNPV to a subsequent generation following the viral infection of *L. dispar* [37,44]. We sterilized egg surfaces before baculovirus DNA detection, suggesting that a significant portion of virus was transmitted to the next generation by the transovarial route. Cabodevilla et al. (2010) [45] showed that horizontally transmitted isolates of Spodoptera exigua multiple nucleopolyhedrovirus strains were significantly more pathogenic than vertically transmitted isolates. They demonstrated that vertically and horizontally transmitted genotypes of the nucleopolyhedrovirus differed in their capability to persist as sublethal infections in the adult stage, thus suggesting that certain vertically transmitted genotypes may be better adapted to vertical transmission. Furthermore, Fuxa and Richter (1991) [46] demonstrated an increased rate of vertical transmission of a strain of Spodoptera frugiperda multiple nucleopolyhedrovirus—a strain that was genetically different from a horizontally transmitted strain [47] and less virulent [48]. In these studies, researchers used strains isolated from pupae or larvae that had been infected through vertical transmission. In the current study, we compared vertical transmission involving two strains of LdMNPV that differed in both genotype and potency [36,49], although both strains were isolated from insects that died following horizontal transmission. In other words, we compared the ability of horizontally infected strains to vertical transmission. We found that both virus strains were transmitted to the next generation, regardless of their virulence.

Many studies have investigated the activation of covert infections in pest populations. Hughes et al., (1993) [22] reported that a culture containing a low-level persistent infection of Mamestra brassicae multiple nucleopolyhedrovirus could be activated when insects were infected by the closely related Panolis flammea multiple nucleopolyhedrovirus and the more distantly related Autographa californica multiple nucleopolyhedrovirus. Activation of covert infection also been shown for other species using a similar approach [50,51,52]. However, Yang et al. (2015) [53] noted that they did not observe activation among covert infections in *L. dispar* larvae by the peroral inoculation of a heterologous virus. Stress factors such as crowded rearing conditions and high relative humidity (RH) were reported to induce higher activation levels of NPVs in the offspring of *Trichoplusia ni* laboratory culture. The activation of *Trichoplusia ni* nucleopolyhedrosis disease was significantly greater at 95% to 100% RH than at RH levels of 75% or less. Crowded rearing conditions activate the covert virus more so than in uncrowded conditions [21]. On the other hand, when using crowding as the stress factor for *L. dispar* larvae, we did not observe the activation of covert infection [54]. Food reduction did not activate covert viral infection of larvae of *Malacosoma pluviale californicum* [25]. Activation of a vertically transmitted virus has been shown using various chemical compounds. After treatment using chemical compounds, mortality varied from 12% to 41% for lethal polyhedrosis disease in covertly infected larvae of *Spodoptera exigua* in the laboratory [55]. Similarly, Ilyinykh et al. (2004) [56] previously reported the activation of LdMNPV in up to 18% of gypsy moth larvae fed on a diet containing 0.6% copper sulphate. Recently we demonstrated activation of the covert virus in an *L. dispar* population using starvation [24]. The mortality rate of more than 70% of the activation of latent infection caused by starvation in covertly infected insects was observed. Thus, different stressing effects may activate the vertically transmitted virus in follow-up generations, allowing the virus to return to horizontal transmission. Our model showed that the mortality induced by starvation indicates that this is an effective factor for NPV activation from covert to overt infection. Interestingly, the majority of dead insects were registered on the first and second days after feeding resumed (i.e., on the fourth and fifth days after the start of starvation). The portion of NPV-specific cadavers in starving groups whose parents were infected with LdMNPV-27/0 (low virulence) was exceeded by about 1.4-fold in comparison with those infected by LdMNPV-45/0 (high virulence). The percentage of spontaneously NPV-induced mortality in OB-treated groups reared without starvation was the same between virus strains. This result indicates the same capability of the virus strains to cause spontaneous polyhedrosis activation, while the effect of the stress factor had a tendency to more effectively activate the low-virulence viral strain. It seems that low virulent strains possess better ability to keep the possibility for transformation from covert to overt form of infection to compare with high virulent strains. It is know that strain of baculoviruses is the mixture of genotypes when it isolated even form single larvae [57]. Thus, we suggest that in low virulent strains, the abundance of genotypes able to successfully overcome/avoid host immunity effect is higher than in high virulent strains.

Many studies have shown debilitating effects on the host after consuming OBs (see review, [58]). In the present study, prolonging the development of infected larvae led to the formation of heavy pupae, although this did not result in increased female productivity since it is usually observed in healthy female *L. dispar* [59]. Rothman and Myers (1994) [60] reported previously that reductions in fecundity might be due to pathological damage to the reproductive tissues of infected females and that there may be no correlation with the pupal mass of insects after OB treatments. The difference in virulence levels of viral strains consumed by parents had some significant effect on parental generation features but did not have an impact on the first generation (i.e., amount of offspring or their survival). Thus, the effectiveness of vertical transmission is the same for low- and high-virulence strains when they are used in the doses that lead to the close effect in mortality. 

The development of highly sensitive molecular tools has enabled researchers to focus their attention on the vertical transmission of insect viruses and to assess the role of vertical transmission in the survival of baculoviruses in both natural and laboratory insect populations [18,19,61,62]. The present findings suggest that low- and high-virulence strains have the same capability to be transferred to the subsequent generation of insects. This demonstrates that surviving insects following NPV application will produce offspring carrying the virus in a covert form. The most interesting property of vertically transmitted baculovirus is its capability to trigger its replication within the host. A portion of insects surviving after treatment is likely to be covertly infected following consumption of OBs and a portion of those survivors may transmit the virus vertically, which is accompanied by subsequent horizontal transmission after the stress factor effect. The study of the capability of different strains for vertical transmission and their capability to reactivate the virus from covert to overt infection allows us to examine these capabilities as an additional strategy for pest control. The possibility of triggering lethal diseases and initiating viral epizootics could improve the effectiveness and reduce the cost of baculovirus-based control methods. This opens widespread perspectives for use in plant protection, especially against pests with high migratory activity or against forest pests in which outbreaks often occur in hard-to-reach areas.

## 5. Conclusions

Our study shows no differences in capability to vertical transmission from parent to filial host generation between low- and high virulent strains of LdMNPV when its used in the doses leaded to same lethal effect. However, low virulent strain tends to be more frequently reactivated from covert to overt form of infection by effect of such stress factor as starvation. From pest management point of view this result indicates that if there is the needs to get prolong effect after baculovirus treatment (i.e., inducing spontaneous epizootic in next herbivore generations) it is reasonably to use low virulent viral strains for the application. Further investigation could be focused on the mechanisms of replication of virus within the host after the stress factors effects.

## Figures and Tables

**Table 1:** Summary of effects of Nucleopolyhedrovirus (NPV) treatment on survivors of *Lymantria dispar.*

Cases	Median Development Time, Days				Mean ± SE Pupal Weight, g	Sex Ratio, % of Male	Mean ± SE Fecundity, Egg	Mean ± SE Fertility, %
	Larvae		Pupae					
	Male	Female	Male	Female				
LdMNPV-45/0	31 (*n* = 119) C	34 (*n* = 93) C	12 (*n* = 119) A	11 (*n* = 93) A	1.06 ± 0.03 (*n* = 215) C	64.6 (*n* = 113) B	129.5 ± 16.6 (*n* = 29) B	37.4 ± 4.8 (*n* = 26) B
LdMNPV-27/0	33 (*n* = 41) B	35 (*n* = 66) B	13 (*n* = 45) A	11 (*n* = 72) A	1.18 ± 0.04 (*n* = 114) B	63.8 (*n* = 72) B	110.2 ± 18.1 (*n* = 15) B	43.4 ± 7.7 (*n* = 15) B
Untreated insects	30 (*n* = 108) A	32 (*n* = 140) A	NA	NA	0.82 ± 0.01 (*n* = 248) A	44 (*n* = 248) A	429.2 ± 56.2 (*n* = 10) A	91.6 ± 2 (*n* = 6) A

Identical letters indicate no significant differences among cases (Dunn’s and Tukey’s post hoc test: *p* > 0.05), NA – Not Available.
